# Physical fitness is related to concentration performance in adolescents

**DOI:** 10.1038/s41598-023-50721-0

**Published:** 2024-01-05

**Authors:** Wolfgang Altermann, Peter Gröpel

**Affiliations:** Division of Sport Psychology, Centre for Sport Science and University Sports, University of Vienna, Vienna, Austria

**Keywords:** Psychology, Human behaviour

## Abstract

The aim of this study was to test the relationship between physical fitness and attention in a sample of adolescents. The hypothesis was that the overall fitness as well as its single components (speed, endurance, strength, coordination, and flexibility) would be positively related to participants’ performance in a test of attention. Participants were adolescent students (*N* = 140) aged 15 to 18 years. Physical fitness was measured with the German Motor Test. Attention was assessed with the d2-Test of Attention. Overall, physical fitness explained 26% of the variance in the attentional test performance. Endurance, strength, coordination, and flexibility were all positively linked to participants’ attention, whereas speed was unrelated to attention. Endurance and flexibility better predicted how fast participants processed the test items, while strength and coordination better predicted the accuracy with which the participants detected the targets. Better physical fitness seems to be an advantage for adolescents’ cognitive performance.

## Introduction

Physical fitness is an important indicator of physical and mental health across the lifespan^1^. An emerging body of research has also shown the importance of physical fitness for cognitive functioning and attention^2,3^. In particular, fitter individuals score in average better on tests of cognition and attention^4–7^, have higher academic performance^8,9^, and gain more cognitive benefits from a physical activity intervention^10–12^. Much of the existing evidence on physical fitness and attention comes from studies with children and adult samples, with fewer studies focusing on adolescents. However, adolescence might be of particular interest because of the possibility to influence neurocognitive maturation of brain regions important for learning-related functions during this period of the lifespan^13,14^.

Adolescence is a sensitive period of maturation, where the brain undergoes rapid development. Prominent developmental transformations are seen in the prefrontal cortex and limbic brain regions, which are critical for cognitive functioning and emotion regulation processes^15,16^. While preadolescents are able to perform tasks that require cognitive skills, fine tuning of cognitive functions continues throughout adolescence until a mature level of performance is achieved in early adulthood. Such fine tuning of cognitive functions is proposed to result from structural and functional changes including synaptic pruning, myelination, and integration of cortical areas^17^. An appropriate cognitive development during adolescence is essential for psychosocial adjustment and learning^9,14,18^, and can be influenced by external stimuli^19^.

In the search for factors that facilitate brain maturation and cognitive performance, researchers repeatedly observed a positive association with physical fitness^3,20,21^. Physical fitness refers to a set of physical attributes to perform physical activities that require speed, endurance, strength, coordination, or flexibility^22^ and is determined by a combination of regular physical activity and genetically inherited ability^1^. Better physical fitness was associated with greater gray matter volume of the hippocampus and the basal ganglia^23,24^, greater white matter microstructure in specific tracts (e.g., the body of corpus callosum; Ref.^25^), and greater thickness of the rostral anterior cingulate cortex^26^, which are all brain structures related to cognitive performance and attentional control. In line with this, the level of physical fitness in adolescence was positively linked to performances on various tests of executive functions^7,21,25^ and attention^6,10,26^.

The majority of studies that showed the relationship between physical fitness and attention have focused on cardio-respiratory fitness (i.e., endurance), with only a few investigations also including other components of physical fitness such as speed, strength, or coordination^3^. Of the few studies that tested several components of physical fitness, Páez-Maldonado et al. found that attentional performance positively correlated with speed and coordination in a pre-adolescent sample, while strength was unrelated to attention^8^. Similarly, Niederer et al. observed that coordination correlated with attentional performance in preschool children^27^. Reigal et al. tested speed, strength, and endurance in a sample of adolescents and found positive correlations of all three fitness components with attentional test performance^6^. Indirect evidence also indicate that several components of physical fitness may be beneficially linked to attention. For example, given that regular physical practice leads to better physical fitness^28,29^, Landrigan et al. reported positive effects of long-term strength training on cognitive functioning in adult samples^30^ and the meta-analytical results of Ludyga et al. imply that coordination training may have even higher cognitive benefits than strength and endurance trainings^31^.

The aim of this study is to test the relationship between physical fitness and attention in a sample of adolescents. Prior evidence indicates that several components of physical fitness, not only endurance, are positively related to attention, yet potentially to a different degree. However, studies that systematically tested the relationship of the five major fitness components (i.e., speed, endurance, strength, coordination, and flexibility) with adolescents’ attention are rare. Such studies are much needed because they might have important implications for school practice. In particular, being aware of which fitness components are most related to adolescent students’ attention may help physical education teachers to decide which types of exercise they should predominantly include in their classes. We thus sampled adolescents aged 15 to 18 years, measured the five major components of physical fitness, and related them to the participants’ attentional performance. We tested both the overall importance of physical fitness as well as the relative importance of each fitness element for participants’ performance in a test of attention. Because overweight and obesity have a negative effect on the development of physical fitness^32,33^, we also assessed participants’ body mass index (BMI) as a potential covariate. We hypothesized that all five components would be positively related to attention.

## Results

Participants’ overall physical fitness (i.e., total Z-score; *M* = 105.13, *SD* = 5.19) was significantly related to attention, as demonstrated by positive correlations with concentration performance score (*r* = 0.485, *p* < 0.001) and speed score (*r* = 0.266, *p* = 0.001) and a negative correlation with error score (*r* = − 0.488, *p* < 0.001). Regarding the single components of physical fitness, almost all of them were significantly correlated with attentional variables (Table 1). BMI was uncorrelated with both physical fitness and attention in our sample, and is thus not discussed any further.

**Table 1:** Descriptive statistics and intercorrelations among study variables (N = 140).

Variables	M	SD	1	2	3	4	5	6	7	8	9
1. BMI percentile	50.56	26.49	–								
Physical fitness											
2. Speed	98.96	9.21	− 0.5	–							
3. Endurance	97.69	9.78	− 0.05	0.31***	–						
4. Strength	107.29	7.56	− 0.06	0.37***	0.14	–					
5. Coordination	108.33	6.56	− .04	0.19*	0.17*	0.46***	–				
6. Flexibility	105.86	8.36	0.01	0.08	− 0.05	0.27**	0.19*	–			
Attention											
7. Concentration score	171.56	30.53	0.02	0.19*0	.24**	0.38***	0.37***	0.31***	–		
8. Speed score	190.17	31.02	− 0.02	0.08	0.18*	0.19*	.18*	0.21*	0.85***	–	
9. Error score	9.67	7.78	− 0.08	− 0.24**	− 0.17*	− 0.40***	− 0.41***	− 0.24**	− 0.44***	0.09	–

BMI percentile = Body Mass Index percentile adjusted for age and gender. Physical fitness scores were Z-transformed using the norming sample with analogous age and gender. Pearson correlation coefficients are reported. By convention, correlations of 0.10, 0.30, and 0.50 are considered small, medium, and large, respectively.

*p <0 .05; **p <0 .01; ***p <0 .001.

Multiple regression analysis revealed that the components of physical fitness altogether explained 26% of the variance in concentration performance score, *R*^*2*^ = 0.26, *F*(5, 139) = 9.55, *p* < 0.001, with all but the speed component significantly predicting attention (Table 2). However, different patterns emerged regarding working speed and error scores in the test of attention. Whereas working speed was significantly predicted by physical endurance (β = 0.17, p = 0.048) and flexibility (β = 0.19, p = 0.033), error score was predicted by physical strength (β = − 0.21, p = 0.025) and coordination (β = − 0.26, p = 0.003). This indicates that participants with more endurance and flexibility worked faster in the test of attention, while those with more strength and better coordination were more accurate in detecting the targets and made fewer errors (Table 2).

**Table 2:** Multiple regression analyses of physical fitness on attention.

	Concentration score			Speed score			Error score		
	*R*^*2*^	β	*t*	*R*^*2*^	β	*t*	*R*^*2*^	β	*t*
Overall model	0.26***			0.10*			0.25***		
Speed		0.01	0.03		− 0.04	− 0.40		− 0.08	− 0.94
Endurance		0.19	2.34*		0.17	2.00*		− 0.08	− 0.97
Strength		0.20	2.22*		0.09	0.95		− 0.21	− 2.27*
Coordination		0.20	2.37*		0.08	0.83		− 0.26	− 3.02**
Flexibility		0.23	2.89**		0.19	2.16*		− 0.13	− 1.67

β = standardized regression weights; *R*^*2*^ = the proportion of variance in the dependent variable explained by the predictor variables.

*p <0 .05; **p <0 .01; ***p < 0.001.

## Discussion

The aim of this study was to test the relationship between physical fitness and attention in a sample of adolescents. In line with the study hypothesis, we found an overall positive association, with physical fitness explaining 26% of the variance in the attentional test performance. Regarding single components of physical fitness, endurance, strength, coordination, and flexibility were all linked to participants’ attention, whereas speed was unrelated to how well participants performed in the test of attention. Detailed analyses further revealed that physical endurance and flexibility better predicted how fast participants processed the test items, while physical strength and coordination better predicted the accuracy with which the participants detected the targets. Better physical fitness thus seems to be an advantage for adolescents’ cognitive performance.

Our results are in line with previous evidence showing that physical fitness is positively associated with cognitive functioning and attention in children and adolescents^2,3,21^ and the related brain structures^23–26^. The explained portion of the variance in attention which we found in the present study corresponds to a large effect (*f*^2^ = 0.351) according to Cohen^34^, implying that higher fitness levels might be beneficial not only for sports, but also for performances that require a great deal of concentration. Indeed, researchers reported that fitter individuals had better academic achievements^8,35^, which has been partly explained by better cognitive functioning^9^. Better physical fitness might thus help adolescents to do well at school and in turn pay off for their career.

Furthermore, our results extend the prior research by simultaneously testing the five major elements of physical fitness (i.e., speed, endurance, strength, coordination, and flexibility; Ref.^22^) and showing that none of the elements is superior to other in predicting attentional performance. Except for physical speed which was unrelated to attention, all other fitness components were significant and similarly high in their magnitude of contribution. However, the single associations were small to moderate and thus any contribution of a single fitness component cannot be overestimated. This observation is in line with prior (limited) evidence showing that several fitness components, not only endurance, may be beneficial for children’s and adolescents’ attentional performance^6,8,27,36^, yet contradicts the notion that coordination training may have higher cognitive benefits than strength and endurance trainings^31^.

Our results also indicate that different elements may be related to different attentional processes. We found that participants with higher physical endurance and flexibility were faster in processing the items in the test of attention, while those with higher physical strength and coordination were more accurate in detecting the targets and made fewer errors. The effect of endurance is in line with a study by Huang et al. who found that higher aerobic fitness (i.e., endurance) in adolescence was associated with shorter reaction times but unrelated to response accuracy in a modified Eriksen flanker task^37^. Similarly, Westfall et al. found that aerobic fitness in adolescence was particularly related to processing speed and information uptake, yet the association with accuracy was also visible^7^. Research on physical activity interventions that mainly included endurance activities demonstrated mixed evidence regarding the trade-off between speed and accuracy in adolescent samples^38^. Regarding flexibility, a greater range of motion may allow people to stay in a position that is less physically taxing and therefore more conducive to focus^39^. Researchers who tested a stretching intervention reported positive effects on selective attention^40^ and better performance in a Stroop task^41^, yet without distinguishing between speed and accuracy. Thus, while our findings indicate higher benefits of physical endurance and flexibility for processing speed than accuracy, the existing evidence is inconclusive and warrants more research in this area.

Physical strength and coordination predicted accuracy rather than processing speed in the test of attention. The association between motor coordination and cognitive accuracy is plausible because they both require various common underlying processes, such as sequencing, monitoring, and planning^42^. Neuroimaging studies with children and adolescents indicate that the brain regions predominantly related to motor activity (cerebellum and basal ganglia) or cognition (the prefrontal cortex) are co-activated during the execution of specific motor activities^43^. Motor coordination especially promotes activation of these areas, which facilitate not only a greater accuracy in motor performance, but also attentional processes controlled by them^44^. Research evidence indeed indicates that motor coordination is linked to better performances in cognitive tasks that are largely based on accuracy^42,45^. Regarding physical strength, previous research is still inconclusive as to whether and why muscular strength is associated with cognitive performance in general, and with accuracy in particular. On the one side, a meta-analysis found positive effects of long-term strength training on cognitive functioning^30^. This may be explained by the release of neurochemicals essential for cognitive processes such as insulin-like growth factor-1^46^ and brain-derived neurotrophic factor^47^, which is promoted by higher muscular fitness^36^. On the other side, other researchers did not find support for a correlation between gross motor skills and cognitive functions in children^42^ or even reported a negative association between upper‐limb absolute strength and accuracy^48^.

Finally, physical speed did not predict participants’ attentional performance. This is at odds with previous studies with children and adolescents showing a positive relationship between physical speed and attentional performance^6,8,27^. This might be due to differences in the physical speed measurement. The previous studies measured speed within an agility test, whereas we used a 20-m linear sprint test to measure participants’ speed. Agility tests (e.g., a shuttle run test, an obstacle course) clearly take speed into account, but other motor skills such as strength and coordination are also included^27^. Consequently, the combination of speed, strength, and coordination rather than speed alone might explain the significant relationship with attention in the previous studies.

The present study has several limitations. First, participants were recruited from a private school which requires tuition fees; consequently, the sample presumably comes from families with higher income. Adolescents from households with higher wealth engage more often in physical exercise outside school, which might have a positive effect on both the participants’ physical fitness and the willingness to take part in the study^49^. Future researchers should sample participants from families with different socio-economic status to enlarge the generalizability of our results. Second, we did not conduct particular analyses for gender and age differences. Our sample size would be too small to include further predictors in the model. However, because we used gender- and age-specific norms to calculate participants’ scores, the scores were comparable for men and women of different ages. Finally, the study design was cross-sectional, which does not allow to set causal relationships. We did not manipulate participants’ fitness level in this study. Although the benefits of long-term physical activity on physical fitness and attention are already well-documented^3^, the question arises whether differential improvements in the five fitness components will have similar differential effects on attentional processes as we documented in this study. This remains an avenue for future research.

In sum, the study results provide evidence for the association between physical fitness and attention in a sample of adolescents. Participants’ endurance, strength, coordination, and flexibility were all linked to their attentional test performance, with a small to moderate magnitude of association. Endurance and flexibility better predicted how fast participants processed the test items, while strength and coordination better predicted the accuracy with which the participants detected the targets. These findings imply that regular physical exercise, which typically increases physical fitness levels^28,29^, should be promoted among adolescent students inside and outside of school setting.

## Method

### Participants and design

This was a preregistered cross-sectional study. An a priori calculation with G*Power^50^ for testing a simple correlation between physical fitness and attention revealed that a sample size of at least 134 persons would give sufficient power (0.95) to detect significant differences at the alpha level of 0.05 with a middle effect size (*r* = 0.30). Participants were students aged 15 to 18 years from an academic secondary school in Vienna, Austria. Inclusion criteria were the target age (15 to 18 years), no physical limitations, and high proficiency in German. For each age category (15, 16, 17, and 18 years), we stopped recruiting when the sub-sample reached 35 persons. The total sample thus included 140 students (69 women and 71 men). The study was conducted in accordance with the Declaration of Helsinki. Participants and their parents signed an informed consent before taking part in the study. The study was approved by the Ethics committee of University of Vienna, Austria (#00595) and ran from February to June 2022.

### Measures

#### Body mass index

Participants’ weight (kg) and height (cm) were measured in sports clothing and barefoot. In particular, body weight was measured with a calibrated digital scale to the nearest 0.1 kg and body height was measured with a stadiometer to the nearest 0.5 cm. Body mass index (BMI) was calculated for every participant as the ratio of body weight to body height squared (kg/m^2^).

#### Physical fitness

Physical fitness was measured with the German Motor Test (GMT), which is a widely used and validated fitness test recommended by the German Society of Sport Science for children and adolescents aged 6 to 18 years^22^. The GMT measures five major components of physical fitness including speed, endurance, strength, coordination, and flexibility.

##### Speed

Speed was assessed with a 20-m sprint, in which participants covered a distance of 20 m from a standing position in as short a time (in seconds) as possible.

##### Endurance

Participants were asked to cover as much distance (in meters) as possible within a 6-min running test.

##### Strength

Participants were asked to perform as many push-ups and sit-ups as possible over a period of 40 s each, and to jump with both legs to reach the greatest possible distance. Sit-ups were performed with hands being placed at the shoulders and the upper body needed to be lifted until the elbows touched the knees. Push-ups were performed from a lying position with hands clasped behind the back to an extended position in which one hand had to be removed from the floor to ensure complete extension of the elbow during each attempt. The standing long jump was performed using a counter-movement jump motion (i.e., swinging the arms) with participants being required to land on their feet and not reaching back with their hands.

##### Coordination

Coordination was assessed with balancing backwards and sideways jumping. Backwards balance was performed across 6 cm, 4.5 cm, and 3 cm wide beams of the same length (300 cm) with participants trying to complete as many steps as possible without a miss. Sideways jumping consisted of jumping back and forth across a line with both legs at the same time for 15 s.

##### Flexibility

Participants completed a stand-and-reach test, in which they stood on a bench and slowly bended forward at the waist, while keeping their knees fully extended. Distance (cm) from the toes was measured, with positive values indicating reaching beyond the toes and negative values indicating not reaching the toes.

#### Attention

Attention was assessed with the revised d2-Test of Attention^51^, which is a widely-used attentional test in school settings due to its simplicity and practicability. The test comprises a single paper sheet with 14 rows of letters (*d*’s and *p*’s) which each are surrounded with up to four short dashes. The task is to search each row of letters, consisting of 57 items, for *d*’s with two dashes and cross them out, while also refraining from responding to seductively similar stimuli (e.g., a *p* with two dashes). Participants were instructed to work as fast and accurately as possible, with a time limit of 20 s per row. Three standard scores—the concentration performance score, the error score, and the speed score—were derived from the test. The concentration performance score is the absolute number of detected targets minus the number of errors, thus reflecting both speed and accuracy. The error score reflects the percentage of incorrectly processed items, either due to omission or commission errors, while the speed score represents the absolute number of detected targets.

### Procedure

#### Participants and their parents signed an informed consent before taking part in the study

Participants were then tested in small groups up to nine persons. They first provided demographics and completed the d2-test of attention. Next, participants’ body weight and height were measured. Thereafter, the GMT was administered, with three test administrators supervising each group of participants. The administrators were familiarized with the test procedures and received a standardized GMT training in advance. Following the exact instruction of the published test manual^22^, the 20-m sprint was performed at the beginning and the 6-min running test was performed at the end of the GMT session. The other GMT subtests were completed in random order in between the running tests; participants moved in a “circle” through subtest stations, with having sufficient time to recover after each subtest, in order to enable time effective testing. Finally, participants were debriefed, thanked, and dismissed.

### Statistical analysis

Values of all GMT subtests were Z-transformed using the norming sample with analogous age and gender, and a total Z-score was calculated as an indicator of participants’ overall physical fitness according to Bös et al.^22^. The physical fitness scores are thus comparable for men and women of different ages. Data are presented as means and standard deviations. Pearson correlations were used to depict the simple relationship between physical fitness, BMI, and attention. BMI percentile adjusted for age and gender was used for data analysis and included as a potential covariate^32,33^. Multiple regression analyses were then conducted to test the relative effect of the five major components of physical fitness on attention. The statistical assumptions associated with regression analysis were met. Normal probability plots of the standardized residual and scatterplots of residuals were generated to test normality, linearity, and homoscedasticity. No serious multicollinearity problems among the predictor variables of the models were found (all variance inflation factor statistics < 4.0). The non-autocorrelation assumption was also met (Durbin-Watson-test; 1.5 < d < 2.5 for all models). All analyses were performed with SPSS 26.0 (IBM Corp.; Armonk, NY, United States). The level of significance was set at *p* < 0.05 (two tailed).

### Ethics statement

The study was carried out in accordance with the Declaration of Helsinki. Participants and their parents signed an informed consent before taking part in the study. The study was approved by the Ethics committee of University of Vienna, Austria (#00595).

## Data Availability

Data are available on figshare (10.6084/m9.figshare.23703276), and pre-registrations of sample size and primary analyses are available on AsPredicted (https://aspredicted.org/blind.php?x=kj3kf8).
